# Environmental filtering shapes microbial mat community assembly across interconnected high-altitude hypersaline systems of the Chilean Altiplano

**DOI:** 10.3389/fmicb.2026.1867952

**Published:** 2026-07-29

**Authors:** María E. Alcamán-Arias, Pablo Vergara-Barros, Karina Fuentes, Daniela Morales, Leslie Vinet, Gustavo Barbosa Athayde, Mario Aguilera, Anelize M. Bahniuk Rumbelsperger, Mauricio Calderón

**Affiliations:** 1 Centro de Investigación en Tecnologías para la Sociedad (C+), Facultad de Ingeniería, Universidad del Desarrollo, Concepción, Chile; 2 Centro de Ciencia del Clima y la Resiliencia (CR2), Concepción, Chile; 3 Departamento de Microbiología, Facultad de Ciencias Biológicas, Universidad de Concepción, Concepción, Chile; 4 Departamento de Ciencias de la Tierra, Facultad de Ciencias Químicas, Universidad de Concepción, Concepción, Chile; 5 Hydrological Research Laboratory (LPH), Universidade Federal do Paraná - UFPR, Curitiba, Brazil; 6 Departamento de Geología, Instituto LAMIR, Universidade Federal do Paraná – UFPR, Curitiba, Brazil

**Keywords:** Atacama-Puna Plateau, ecological connectivity, high-altitude saline ecosystems, microbial community assembly, salinity gradient

## Abstract

High-altitude saline ecosystems of the Atacama–Puna Plateau represent natural laboratories for investigating microbial adaptation and community assembly under polyextreme conditions. Despite their ecological importance, integrated assessments of microbial diversity across contrasting Andean systems remain scarce. Here, we characterized microbial mats, sediments, and saline crusts across three high-altitude sub-basins of northern Chile, including the Maricunga Salt Flat–Laguna Santa Rosa hydrological system, Laguna del Negro Francisco, and Laguna Verde. Microbial communities were analyzed using 16S rRNA gene amplicon sequencing, environmental characterization, predicted metabolic profiling, and co-occurrence network analyses. Salinity was the only environmental variable significantly associated with microbial community composition, although site identity explained a greater proportion of variation, highlighting the influence of local environmental conditions. Across ecosystems, communities were dominated by Pseudomonadota and Bacteroidota, whereas hypersaline habitats were enriched in halophilic archaeal lineages and brackish environments exhibited greater representation of photosynthetic and nitrogen-related functions. Only 15% of ASVs were shared between brackish and hypersaline habitats, indicating strong habitat specialization. Nevertheless, co-occurrence networks revealed a small set of highly connected keystone taxa shared among ecosystems, forming a regional core microbiome. Together, these findings demonstrate that salinity promotes taxonomic and functional specialization, while hydrological connectivity maintains ecological cohesion across interconnected ecosystems. By integrating lagoons, wetlands, saline crusts, and geothermal habitats within a regional framework, this study provides the first regional-scale assessment of microbial diversity, community assembly, and ecological connectivity across the southern Atacama–Puna Plateau.

## Introduction

The southern Chilean sector of the Altiplano-Puna Plateau, in the Atacama Region, hosts a network of high-altitude Andean saline lakes and salt-flats exposed to some of the most extreme environmental conditions on Earth. These environments are characterized by intense ultraviolet (UV) radiation, hypersalinity, low atmospheric pressure, high evaporation rates, and wide diurnal temperature fluctuations (Demergasso et al., 2010; Dorador et al., 2013; Farías et al., 2017; Gajardo and Redón, 2019; Ben Abdallah et al., 2024). These polyextreme settings impose strong physicochemical constraints that shape microbial life, favoring highly specialized communities adapted to osmotic stress, radiation, and thermal variability (Castro-Severyn et al., 2021; Kurth et al., 2021; Pardo-Esté et al., 2023). Key systems within this region include the Maricunga Salt Flat (~ 3,760 m a.s.l.), Laguna Santa Rosa (~ 3,760 m a.s.l.), Laguna del Negro Francisco (~ 4,125 m a.s.l.), and Laguna Verde (~ 4,300 m a.s.l.). The Maricunga Salt Flat hosts both surface and deep brines and evaporitic crusts with significant concentrations of lithium and other critical elements and is hydrologically connected to the Ramsar-designated Laguna Santa Rosa through the Maricunga Wetland Main Channel, forming an integrated hydrological and geochemical system (Alam and Sepúlveda, 2022). Such connectivity generates steep environmental gradients that may structure microbial assemblages across these systems (Kimbrel et al., 2018). Laguna del Negro Francisco is divided into two hydrological compartments with contrasting salinity by an alluvial ridge: an eastern open brackish lagoon and a western hypersaline lagoon (Grosjean et al., 1997; Risacher et al., 1999). This internal spatial heterogeneity creates contrasting salinity regimes within a single hydrological unit, providing a natural framework to explore how ionic composition and osmotic stress influence microbial diversity, community turnover, and functional potential across short spatial scales. Laguna Verde exemplifies intensified high-altitude environmental conditions combining pronounced thermal variability in hot springs (15–47 °C) with hypersaline lagoon conditions, where salinity reaches 70–80 g L^−1^ (Alam and Muñoz, 2024). These contrasting environmental conditions offer a unique natural laboratory to examine how salinity, temperature, and hydrological isolation interact to influence microbial community assembly, spatial structuring, and adaptive responses across interconnected polyextreme ecosystems.

Across these Andean polyextreme environments, hydrological connectivity and steep geochemical gradients promote the establishment of diverse microbial habitats, including stratified, metabolically interdependent communities organized into organo-sedimentary structures known as microbial mats, as well as sediment-associated microbial layers and evaporitic salt crusts inhabited by halotolerant and halophilic microorganisms. Together, these microhabitats represent distinct ecological niches shaped by the extreme physicochemical conditions characteristic of high-altitude salt flats. Beyond their structural complexity microorganisms play a fundamental role in the functioning of these ecosystems, as their metabolic diversity directly drives biogeochemical cycling, primary productivity, and mineral precipitation processes (Gomez et al., 2014; Castro-Severyn et al., 2021; Kurth et al., 2021; Osman et al., 2024). Across microbial mats and endoevaporitic salt crusts, microbial communities are typically dominated by members of the phyla Pseudomonadota, Bacteroidota, Cyanobacteriota, and Chloroflexota. These bacterial groups are frequently accompanied by abundant Euryarchaeota, especially in highly evaporitic and lithified environments where archaeal populations may equal or even exceed bacterial abundance (Farías et al., 2017; Vignale et al., 2021; Osman et al., 2024). Despite pronounced taxonomic variability, the functional potential of these communities frequently shows convergent patterns, especially in metabolic pathways related to carbon fixation, sulfur and methane cycling, and arsenic metabolism. Biogeochemical profiles further indicate that microbial activity tends to decrease with increasing salinity and degree of lithification, highlighting salinity as a key environmental driver shaping biodiversity and metabolic intensity in these systems (Fernandez et al., 2016; Kurth et al., 2021; Pardo-Esté et al., 2023). A previous study conducted within our study area have provided the first insights into the microbial diversity inhabiting these high-altitude ecosystems, although they remain largely exploratory and geographically restricted (Borsodi et al., 2022). In sediments from Laguna Santa Rosa, Bacteroidota (mainly Flavobacteriaceae and Cryomorphaceae) and Firmicutes (Halanaerobiaceae) have been reported as dominant taxa. In contrast, microbial mats associated with the hot springs of Laguna Verde were characterized by the predominance of Cyanobacteriota, Chloroflexota (primarily Anaerolineaceae), and Planctomycetota (Borsodi et al., 2022). However, this pioneering study focused on individual habitats or specific locations, providing only a fragmented view of microbial diversity across the Altiplano-Puna region. Consequently, the spatial distribution of microbial communities along interconnected environmental gradients, and the extent to which salinity, temperature, hydrological connectivity, and local geochemistry shape community assembly, remain poorly understood.

Despite the ecological and geochemical importance of these high-altitude Atacama-Puna systems, integrated evaluations of microbial diversity across Laguna Santa Rosa, Maricunga Wetland Main Channel, Maricunga Salt Flat, Laguna del Negro Francisco, and Laguna Verde remain scarce. Understanding how microbial communities are distributed across these naturally connected yet environmentally contrasting habitats is fundamental for revealing the ecological processes that structure biodiversity in Andean polyextreme ecosystems. Such knowledge is particularly relevant under increasing pressures from lithium extraction and climate-driven hydrological change, which may alter the environmental gradients sustaining these unique microbial assemblages. Establishing a robust baseline of microbial diversity and community assembly across these basins will therefore improve our understanding of ecosystem functioning, resilience, and vulnerability.

## Materials and methods

### Sampling site

**Figure 1:**
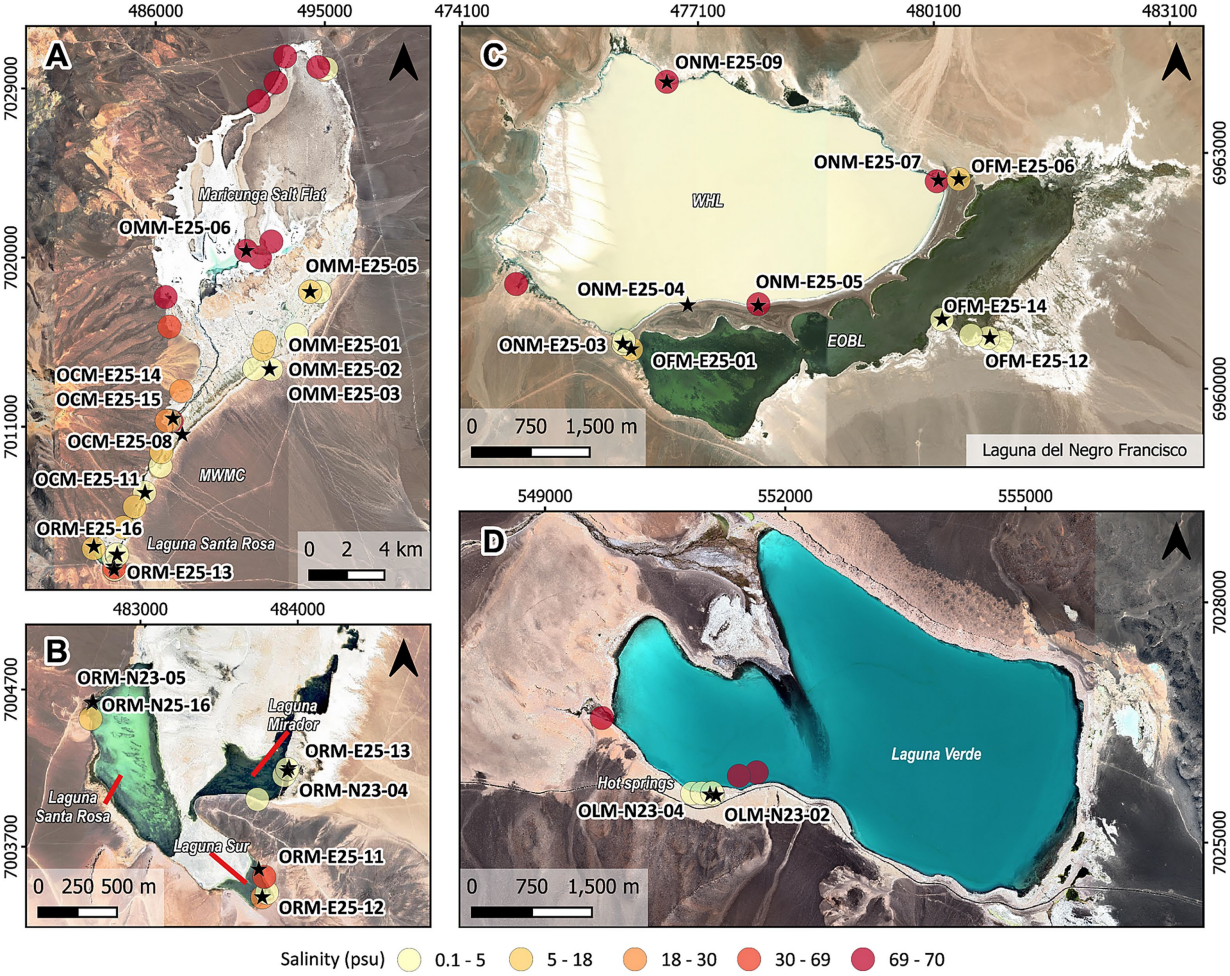
Geographic distribution of microbial sampling sites. **(A)** Overview map of the Maricunga Salt Flat, the Maricunga Wetland Main Channel (MWMC), and Laguna Santa Rosa. **(B)** Detailed map of Laguna Santa Rosa, including Laguna Sur, Laguna Mirador, and Laguna Santa Rosa. **(C)** Laguna del Negro Francisco, including the Eastern Open Brackish Lagoon (EOBL) and Western Hypersaline Lagoon (WHL). **(D)** Geothermal springs of Laguna Verde. Samples were collected during field campaigns conducted in November 2023 (N23) and January 2025 (E25). Colored circles represent salinity values according to the corresponding scale bar, highlighting the spatial variability of salinity across interconnected brackish, hypersaline, and geothermal environments.

The study area is located in the Atacama-Puna of northern Chile, within the southern sector of the Altiplano-Puna Plateau in the Central Andes. This area corresponds to the southern hydrological system of the Maricunga Salt Flat Basin, which comprises a network of interconnected ecological units (Figures 1A,B). In the southern sector, a lagoon complex formed by Laguna Sur, Laguna Santa Rosa, and Laguna Mirador (Figure 1B) is connected to the central Maricunga Salt Flat by the Maricunga Wetland Main Channel (MWMC), a permanent, slow-flowing channel that transports water northward (Figure 1A). Downstream, the system drains into the Maricunga Salt Flat, whose central basin is characterized by extensive halite (NaCl) and sulfate (CaSO₄) deposits interspersed with brackish and hypersaline lagoons. These water bodies exhibit elevated concentrations of sodium, chloride, sulfate, potassium, and lithium, with values generally exceeding the third quartile relative to all other sampled hydrological environments, including hydrothermal vents, drainage channels, and lagoons. The salt flat also receives additional hydrological inputs from several tributary streams and groundwater discharge originating in the surrounding Andean catchments. Additional monitored sites included saline lagoons such as Laguna del Negro Francisco and Laguna Verde. Laguna del Negro Francisco (Figure 1C) is characterized by the presence of two distinct water bodies, locally referred to as the Eastern Open Brackish Lagoon (EOBL) and Western Hypersaline Lagoon (WHL), which form a single endorheic system separated by a low alluvial ridge. Geologically, the area is surrounded by Miocene volcanic centers that display extensive pyrite-dominated epithermal alteration zones, associated with significant gold mineralization in the surrounding volcanic complexes. The other study site, Laguna Verde (Figure 1D) is located within the Andean volcanic chain, where geological records indicate volcanic and hydrothermal activity since the Pleistocene. The area currently exhibits active geothermal manifestations, including hot springs that support extensive microbial mat communities characteristic of hydrothermal ecosystems, highlighting Laguna Verde as a representative polyextreme environment.

Fieldwork for environmental data collection and microbial community sampling was conducted during two field campaigns, in November 2023 (N23) and January 2025 (E25). These surveys covered the main ecological units of the system including: two sampling points from Laguna Sur (ORM-E25-11 and ORM-E25-12), Laguna Mirador (ORM-N23-04 and ORM-E25-13) and Laguna Santa Rosa (ORM-N23-05 and ORM-E25-16), and five sampling points along the MWMC (OCM-E25-08, OCM-E25-11, OCM-E25-14_1, OCM-E25-14_2, OCM-E25-15), and Maricunga Salt Flat (OMM-E25-01, OMM-E25-02, OMM-E25-03, OMM-E25-05, OMM-E25-06_A). At Laguna del Negro Francisco, sampling sites were distributed between four locations in the Eastern Open Brackish Lagoon (EOBL) (OFM-E25-01, OFM-E25-06, OFM-E25-12, OFM-E25-14) and five locations in the Western Hypersaline Lagoon (WHL) (ONM-E25-03, ONM-E25-04, ONM-E25-05, ONM-E25-07, ONM-E25-09). At Laguna Verde, two additional sampling points in hot springs were selected (OLM-N23-02 and OLM-N23-04_G).

At each sampling point, *in situ* physicochemical parameters were measured, including temperature (°C), pH, conductivity (mS/cm), Total Dissolved Solids (ppt), salinity (psu), and dissolved oxygen (ppm), using a portable multiparameter probe (Hanna Instruments HI98194) (Supplementary Table S1). Microbial mats, layered microbial sediments, and endoevaporitic salt crusts were collected arbitrarily at each sampling site using a sterile stainless-steel spatula and transferred into 15 mL sterile polypropylene tubes in triplicate. For each replicate, 1–2 mL of RNAlater™ stabilization solution was added and gently homogenized to preserve the biological material. Samples were then frozen at −20 °C and stored under these conditions until laboratory processing.

### Environmental DNA extraction and sequencing

To obtain total environmental DNA from microbial mats, layered sediments, and saline crusts, approximately 30 mg from each replicate were processed using the organic phenol–chloroform extraction method previously described by Alcamán-Arias et al. (2018), with minor modifications. Prior to cell lysis, samples were subjected to three washes with 1X phosphate-buffered saline (PBS) followed by a final wash with nuclease-free water to remove excess salts and humic compounds characteristic of these matrices, according to the adapted processing protocol for saline samples described by Farías et al. (2014). Subsequently, chemical lysis was performed using a buffer composed of potassium ethyl xanthogenate, ammonium acetate, Tris–HCl, and ethylenediaminetetraacetic acid (EDTA), followed by mechanical lysis through bead beating with 0.1 mm diameter glass beads (Mini-BeadBeater-1.6, Model 607EUR, BioSpec Products). Thereafter, sodium dodecyl sulfate (SDS), lysozyme, and proteinase K were added, and samples were incubated at 65 °C for 2 h. DNA was eluted in 100 μL of nuclease-free water, and extracts from the same sampling site were pooled prior to storage at −20 °C until further analysis.

The quality of the extracted DNA was assessed by measuring the 260/280 ratio using an Epoch™ microplate spectrophotometer (BioTek Instruments) with Gen5™ software. The presence of amplifiable bacterial DNA and the absence of PCR inhibitors were evaluated by PCR amplification using universal primers targeting the bacterial 16S rRNA gene (358F and 907R). PCR products were visualized by electrophoresis on a 1% agarose gel to confirm successful amplification of the 16S rRNA gene and to assess the integrity of the extracted DNA. DNA samples were lyophilized using Alpha 1–2 LO Plus equipment and sent to Argonne National Laboratory (IL, United States) at room temperature. 16S rDNA Libraries for each sample were constructed using 515F–806R primer pair (Caporaso et al., 2011) using a commercial kit and sequenced using Illumina MiSeq (Illumina, San Diego, CA) (Caporaso et al., 2011).

### Taxonomic assignment of 16S rRNA sequences

Illumina-obtained 16S sequences were demultiplexed and cleaned from primers and adapters using QIIME2 v2025.7 and cutadapt v5.1 (Martin, 2011; Bolyen et al., 2019). Additional sequences quality filter and trimming were done using FASTP v1.0.1 (Chen et al., 2018). Amplicon Sequence Variants (ASV) were selected and quantified using QIIME2 and dada2 command denoise-paired (Callahan et al., 2016) and the taxonomic identity of the representative ASV was assigned to GreenGenes2 database v2024.09 (McDonald et al., 2024) using option classify-sklearn from the q2-feature-classifier plugin (Bokulich et al., 2018). Taxa groups which were divided into different subgroups denoted by letters or numbers were regrouped into one group (e.g., Bacillota_A_368345 and Bacillota_B_370514 into Bacillota).

### Taxonomic abundance and diversity analysis

The ASVs abundance and respective taxonomic annotations were analyzed in R using the package ampvis 2 v2.7.31 (Andersen et al., 2018), along with the metadata from the corresponding samples. Samples with fewer than 1,000 sequences were discarded. To obtain phyla and orders abundance, data was normalized using ampvis2 and tabulated using R’s package tidyverse (Wickham et al., 2019). Also, alpha diversity was performed using ampvis2’s amp_alpha_diversity() command to calculate Shannon and Chao1 indexes. For statistical analysis of the alpha diversity differences between samples, we used the statistic tests Welch’s ANOVA for multiple groups comparison, followed by Tukey *post-hoc* test for multiple groups post-hoc paired comparisons using the program ggstatsplot v0.13.5 (Patil, 2021). For the Beta diversity analysis, we constructed RDA projections using ampvis2’s amp_ordinate() command constrained against the samples, and environmental variables were fitted as vectors within the RDAs. Also, correlation between normalized phyla abundance and environmental variables was calculated using rcorr command from R’s Hmisc package v5.2–1 (February 26, 2026).^1^

### Environmental characterization and variable selection

To characterize the environmental conditions of the different sectors evaluated, physicochemical variables were first analyzed independently from the microbial community sampling. Pairwise correlations among environmental variables were assessed using the cor.test() function in R, and highly correlated variables were excluded to avoid multicollinearity in subsequent analyses. Because environmental measurements were not directly paired with individual microbiome samples, the environmental dataset was used to identify the main physicochemical gradients across sampling sectors. To determine which environmental variables were significantly associated with microbial community composition, a forward selection procedure was performed using the ordiR2step() function from the R package vegan (Dixon, 2003). Bray–Curtis dissimilarities calculated from 16S rRNA gene amplicon data were compared with environmental variables, and only variables that significantly improved model performance were retained. The selected variables were subsequently used to characterize environmental differences among sectors and to evaluate their contribution to microbial community structuring.

### Microbial co-occurrence network analysis

To evaluate microbial co-occurrence patterns, correlations among ASVs identified from 16S rRNA gene sequencing data were analyzed. To reduce the potential inflation of correlations caused by the high proportion of zero values typically present in microbiome datasets, a two-layer network approach was implemented by separating ASVs into core and rare fractions. Core ASVs were defined as those present in more than 20% of samples, whereas rare ASVs were defined as those occurring in 20% or fewer samples.

For the core layer, associations among core ASVs were inferred using the SPIEC-EASI algorithm implemented in the R package SpiecEasi (Kurtz et al., 2015) applying the Meinshausen–Bühlmann neighborhood selection method (Meinshausen and Bühlmann, 2010). For the rare layer, Spearman’s rank correlations were calculated between rare and core ASVs using the cor.test() function in R. Only positive associations with a correlation coefficient (*ρ*) > 0.6 and a *p* < 0.01 were retained. The resulting positive core-to-core and rare-to-core association matrices were merged to construct the final microbial co-occurrence network, which was visualized in Cytoscape (Shannon et al., 2003). Network topology was subsequently analyzed using the Cytoscape Analyze Network function. Putative keystone ASVs were identified based on node degree, with highly connected nodes considered to have a potentially important role in maintaining network structure and connectivity.

### Predicted metabolic profiling

To estimate the metabolic potential associated with biogeochemical cycling, predicted functional profiles were generated using FAPROTAX v 1.2.12 (Louca et al., 2016). As FAPROTAX database is based on the SILVA SSU taxonomy (v132) (Glöckner et al., 2017; Chuvochina et al., 2026), ASVs were taxonomically reassigned against the SILVA SSU database following the same procedure previously applied using GreenGenes2 v2024.09. Predicted metabolic functions were subsequently inferred for each sample from the SILVA-assigned ASV count table using the collapse_table.py script implemented in FAPROTAX. The resulting functional profiles were analyzed and visualized in R.

## Results

### Environmental characterization and drivers of microbial community structure

**Figure 2:**
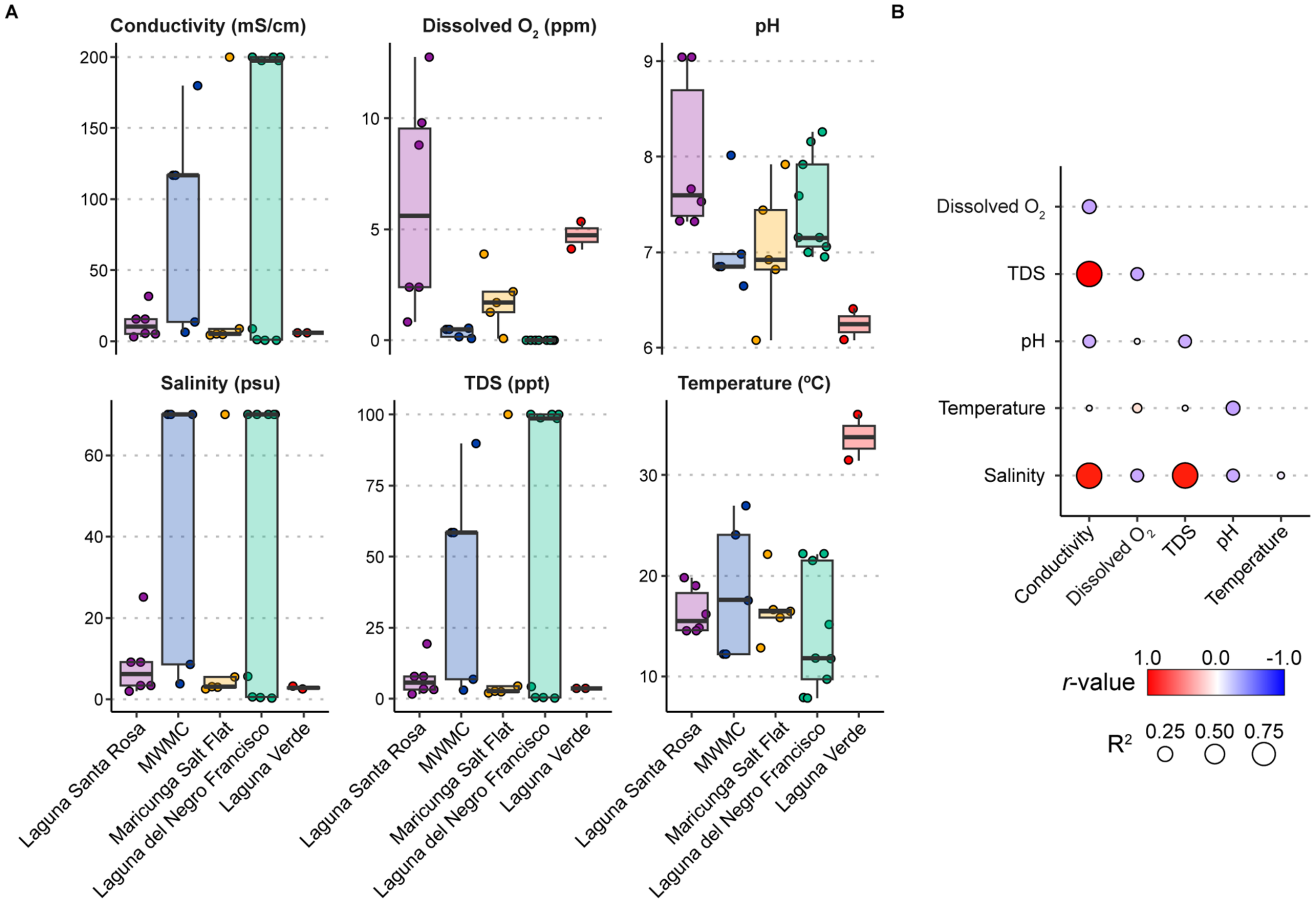
Environmental characterization of the studied ecosystems. **(A)** Distribution of physicochemical variables measured across Laguna Santa Rosa, the Maricunga Wetland Main Channel (MWMC), the Maricunga Salt Flat, Laguna del Negro Francisco, and Laguna Verde, including conductivity (mS/cm), dissolved oxygen (ppm), pH, salinity (psu), total dissolved solids (TDS, ppt), and temperature (°C). Boxplots represent the variability of measurements within each ecosystem, with points indicating individual sampling locations. **(B)** Correlation matrix of environmental variables. Circle color indicates the direction and strength of the correlation coefficient (r), whereas circle size represents the proportion of explained variance (R^2^).

The physicochemical characterization revealed pronounced environmental heterogeneity among the five monitored sectors, defining distinct ecological niches across the studied system (Figure 2; Supplementary Table S1). Conductivity, salinity, and TDS showed highly similar spatial patterns, with the highest values recorded in WHL, the northern sector of the MWMC, and the hypersaline lagoon of the Maricunga Salt Flat (Supplementary Table S1). In contrast, Laguna Santa Rosa, the central and southern sector of MWMC, and Laguna Verde maintained comparatively diluted conditions (Figure 2A). In contrast, dissolved oxygen tended to decrease as salinity and conductivity increased, with near-anoxic conditions observed (0–0.45 ppm) in the most saline environments (Supplementary Table S1), whereas Laguna Sur displayed the highest oxygen concentrations (9.79 ppm). Temperature clearly distinguished Laguna Verde from the remaining sites, where geothermal activity-maintained values between 31 and 36 °C. Although most sites were generally alkaline, pH showed variation from 6.08 to 9.04 (Supplementary Table S1) and showed weak correlations with the other environmental parameters. The boxplots (Figure 2A) show both the central tendency and variability of each parameter, whereas the correlation matrix summarizes the strength (R^2^, circle size) and direction (*r*-value) of the relationships among variables (Figure 2B). Correlation analysis further confirmed that conductivity, salinity, and TDS were strongly positively correlated, suggesting that ionic concentration represents a major environmental gradient structuring the system (Supplementary Table S1). The combination of these environmental gradients likely constitutes the main selective force shaping microbial community composition and ecological differentiation across the studied system. To further evaluate the relative contribution of these environmental factors to microbial community organization, a variation partitioning analysis was performed (Supplementary Table S2). When considering only physicochemical parameters, salinity was the only variable that significantly explained variation in community composition (adjusted R^2^ = 0.027, *p* = 0.024), supporting its role as an important ecological filter across these high-altitude systems. Temperature, pH, and dissolved oxygen individually explained larger proportions of variance, but none of their effects reached statistical significance. The inclusion of site identity improved the explanatory power of the model, with site accounting for 7.0% of the variation in community composition (adjusted R^2^ = 0.070, *p* = 0.050). Under this model, salinity (adjusted R^2^ = 0.096, *p* = 0.063) and temperature (adjusted R^2^ = 0.122, p = 0.063) explained a greater proportion of variation than in the physicochemical-only model, although neither variable remained statistically significant. Nevertheless, both variables showed a marginal trend, suggesting that their influence on community structure may be partially associated with site-specific environmental conditions. This pattern is particularly evident for temperature, likely reflecting the distinctive geothermal conditions of Laguna Verde rather than a generalized thermal gradient across the entire hydrological network. In contrast, pH remained non-significant (*p* = 0.280), indicating a limited contribution to microbial community differentiation (Supplementary Table S2).

### Diversity, community assembly, and taxonomic composition across high-altitude saline ecosystems

**Figure 3:**
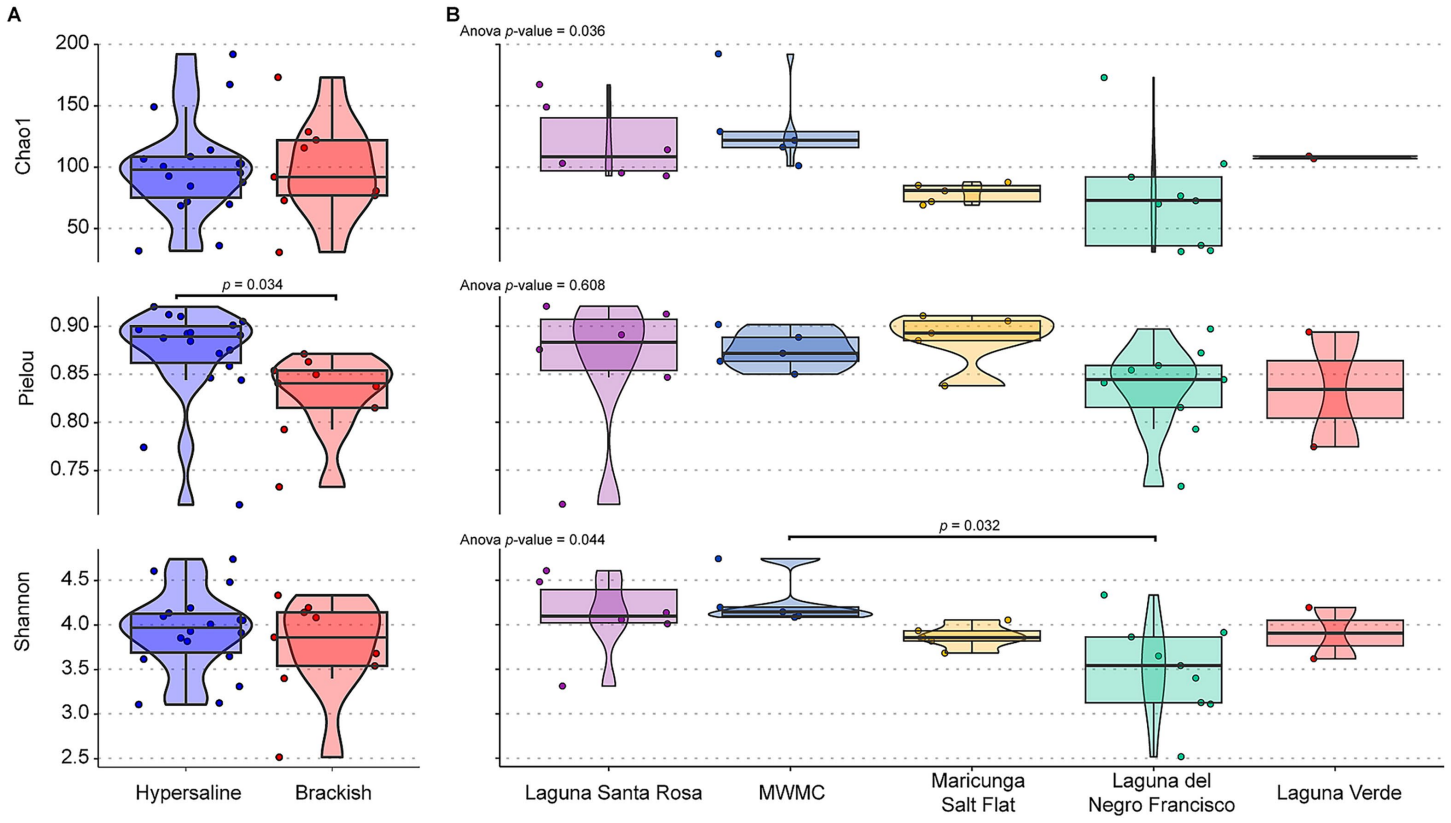
Alpha diversity of microbial communities. **(A)** Comparison of Chao1 richness, Pielou evenness, and Shannon diversity indices between brackish and hypersaline environments. **(B)** Comparison of the same alpha-diversity indices among ecosystems, including Laguna Santa Rosa, the Maricunga Wetland Main Channel (MWMC), the Maricunga Salt Flat, Laguna del Negro Francisco, and Laguna Verde. Violin plots represent the distribution of values, boxplots indicate the median and interquartile range, and points correspond to individual samples. *p*-values are shown in the upper-left corner of each panel.

Microbial alpha diversity showed limited variation across the environmental gradient (Figure 3). Given that salinity was identified as a key environmental filter across the study area, samples were first grouped into two broad categories, hypersaline and brackish, and subsequently analyzed according to their specific site locations to assess finer-scale spatial patterns in diversity. When samples were grouped by salinity (Figure 3A), Chao1 richness and Shannon diversity did not show significant differences between hypersaline and brackish environments. However, Pielou evenness was significantly higher (*p* = 0.034), suggesting a more even distribution of taxa under high-salinity conditions. In contrast, grouping samples according to geographic location revealed significant differences in overall community diversity (ANOVA, *p* = 0.044) (Figure 3B). Pairwise comparisons showed that the MWMC exhibited significantly higher Shannon diversity than Laguna del Negro Francisco (*p* = 0.032), whereas no significant pairwise differences were detected for Chao1 richness or Pielou evenness.

**Figure 4:**
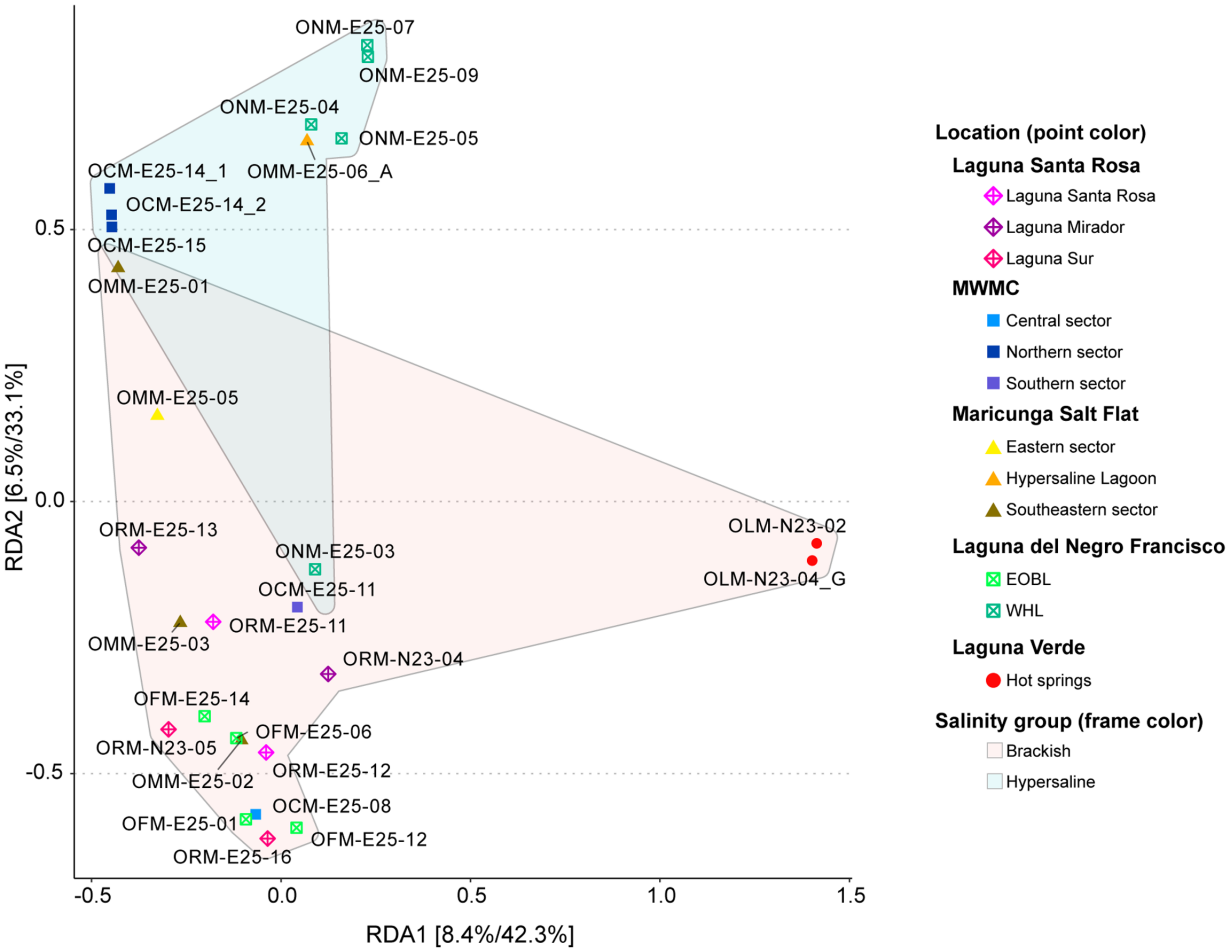
Redundancy analysis (RDA) of microbial communities based on Bray–Curtis dissimilarities. Samples are colored according to their ecosystem of origin, including Laguna Santa Rosa (Laguna Sur, Laguna Mirador, and Laguna Santa Rosa), the Maricunga Wetland Main Channel (MWMC), the Maricunga Salt Flat, Laguna del Negro Francisco [Eastern Open Brackish Lagoon (EOBL) and Western Hypersaline Lagoon (WHL)], and Laguna Verde. Polygon shading indicates salinity groups (brackish and hypersaline). Each point represents an individual sample, and sample labels correspond to field collection identifiers. The percentages shown on the axes indicate the proportion of constrained and total variance explained by each RDA axis.

Beta diversity analysis revealed marked differences in microbial community composition across the environments studied (Figure 4). The first two RDA axes explained 14.9% of the total variation, accounting for 42.3 and 33.1% of the constrained variation, respectively. The most distinct pattern corresponded to Laguna Verde hot springs, whose samples formed an isolated cluster along positive RDA1 values, indicating a unique community composition relative to all other sites. This segregation likely reflects the distinctive geothermal conditions and elevated temperatures of Laguna Verde compared with the rest of the hydrological system. In contrast, Laguna Santa Rosa, the MWMC (central and southern sector), and the Maricunga Salt Flat (eastern and southeastern sector) exhibited substantial overlaps, suggesting strong compositional similarity among these hydrologically connected environments. Samples from Laguna del Negro Francisco (EOBL) occupied an intermediate position and displayed greater internal dispersion toward the hypersaline group (northern sector), consistent with the coexistence of brackish and hypersaline habitats within the system. Although hypersaline and brackish samples showed a tendency to segregate, the substantial overlap between salinity groups indicates that salinity alone does not fully explain community differentiation. Instead, beta-diversity patterns suggest that site-specific environmental conditions, together with hydrological connectivity, play an important role in structuring microbial mat communities across the study area (Figure 4).

**Figure 5:**
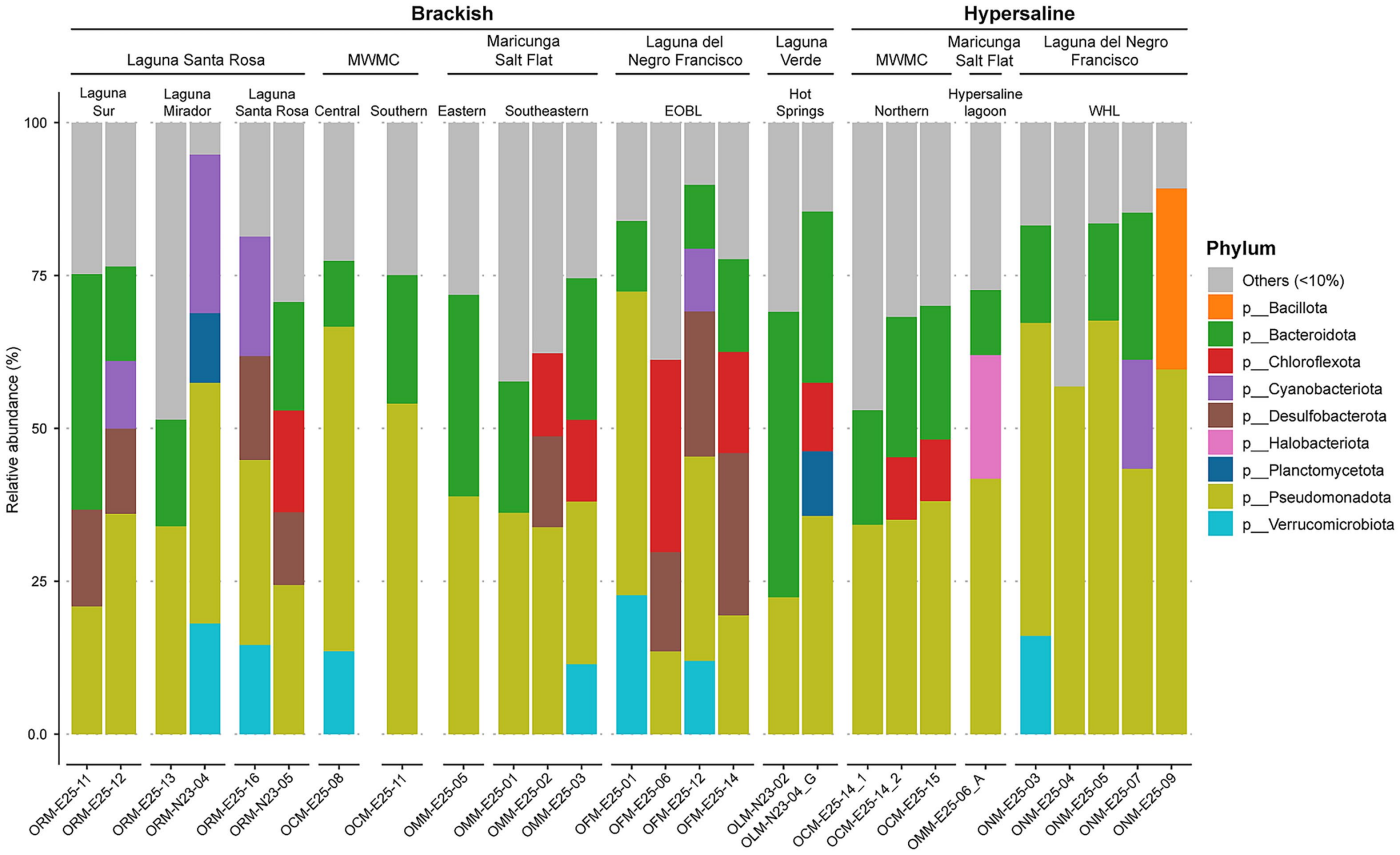
Taxonomic composition of microbial communities at the phylum level across the studied ecosystems. Stacked bar plots show the relative abundance (%) of phyla representing more than 10% of the community in at least one sample. Samples are grouped according to ecosystem and salinity category (brackish and hypersaline), including Laguna Santa Rosa, the Maricunga Wetland Main Channel (MWMC), the Maricunga Salt Flat, Laguna del Negro Francisco, and the geothermal springs of Laguna Verde. Colors indicate different microbial phyla, while the gray category (“Others”) comprises all phyla with relative abundances below 10%.

Taxonomic assignment of 16S rRNA gene sequences at the phylum level, considering taxa with relative abundances greater than 10%, revealed diverse microbial communities across all sampling sites. Despite variation in relative abundance among subsamples, the communities were consistently dominated by the phyla Bacteroidota and Pseudomonadota (Figure 5). Within the brackish group, the three monitored lagoons associated with Laguna Santa Rosa: Laguna Sur (ORM-E25-11 and ORM-E25-12), Laguna Mirador (ORM-N23-04 and ORM-E25-13), and Laguna Santa Rosa (ORM-N23-05 and ORM-E25-16) exhibited distinct taxonomic profiles that varied both among lagoons and between sampling years. These differences were primarily reflected in shifts in the relative abundance of dominant phyla, indicating that local environmental conditions and temporal variability influence community composition even within the same hydrological complex. Laguna Sur was characterized by the dominance of Bacteroidota (38.56%) and Pseudomonadota (36.02%), whereas Desulfobacterota (15.83%) and Cyanobacteriota (11.07%) were present at lower relative abundances. In contrast, Laguna Mirador and Laguna Santa Rosa, which were sampled in both 2023 (N23) and 2025 (E25), displayed pronounced interannual changes in community composition while consistently maintaining Pseudomonadota as the dominant phylum, reaching relative abundances of up to 39.37%. The observed temporal variation was mainly associated with fluctuations in the abundance and occurrence of Bacteroidota, Cyanobacteriota, and Planctomycetota. Notably, Laguna Santa Rosa exhibited the highest taxonomic complexity among the brackish lagoons, being the only site where both Desulfobacterota and Chloroflexota were detected. In the MWMC, Pseudomonadota (up to 54%), Bacteroidota (up to 21%), and Verrucomicrobiota (up to 13%) were the most represented phyla. The Eastern and Southeastern sectors of Maricunga Salt Fat also showed that Pseudomonadota reached up to 38.86% and was mainly associated with saline crust and presence of oncolites. In turn, Bacteroidota reach >32.99% of dominance, being the Chloroflexota (13.6%) and Desulfobacterota (14.87%) the most prevalent. Laguna del Negro Francisco displayed the most distinctive community composition among the sampled sites, characterized by pronounced shifts in the relative abundance of dominant phyla between the brackish and hypersaline sectors. This pattern contrasted with the more stable taxonomic structure observed in the remaining locations. Notably, the brackish lagoon was enriched in Chloroflexota and Desulfobacterota, which reached relative abundances of 31.50 and 26.54%, respectively, together with Verrucomicrobiota (22.70%). In contrast, these phyla markedly decreased or were no longer detected in the hypersaline lagoon, where the community was dominated by Pseudomonadota (up to 67.61%). These results suggest a strong influence of local salinity conditions on community composition within the Laguna del Negro Francisco system. Together with the other hypersaline environments, Pseudomonadota was the dominant phylum in both the northern sector of the MWMC and the saline crusts of the Maricunga Salt Flat (average of 37.27%). In addition, Halobacteriota emerged as a characteristic component of the salar crust communities, reaching relative abundances of up to 20.24% (Figure 5). This pattern highlights the importance of archaeal populations under extreme salinity conditions, where members of Halobacteriota are known to thrive. Lower abundances of this phylum were also detected in the hypersaline crusts of Laguna del Negro Francisco (9.19%), suggesting that archaeal taxa constitute a common feature of the most saline habitats within the studied area. Laguna Verde hot springs were dominated by Bacteroidota and Pseudomonadota, although their relative abundances varied along the thermal gradient. Bacteroidota decreased from 46.70% at 36 °C to 28.03% at 31.5 °C, while Pseudomonadota declined from 35.66 to 22.36% across the same temperature range. Conversely, Chloroflexota (11.19%) and Planctomycetota (10.58%) became more prominent at 31.5 °C, indicating that slightly lower temperatures support a more diverse taxonomic assemblage within the geothermal habitat.

Across the dominant phyla, lower taxonomic ranks exhibited remarkable consistency across sites. Bacteroidota was primarily represented by the orders Bacteroidales and Flavobacteriales, whereas Pseudomonadota was dominated by Rhodobacterales, Sphingomonadales, Burkholderiales, and Chromatiales. Cyanobacteriota was mainly associated with Cyanobacteriales and the PCC-6307 lineage, while Verrucomicrobiota was represented predominantly by Verrucomicrobiales. Desulfobacterota was characterized by the orders Desulfobacterales and Desulfobulbales, Chloroflexota by Anaerolineales, and Bacillota by Halanaerobiales (Supplementary Figure S1). Despite marked differences in phylum-level relative abundances among sites, these recurrent taxonomic groups were consistently detected across the study area, suggesting the presence of conserved ecological lineages adapted to the physicochemical conditions of high-altitude saline ecosystems.

### Taxonomic shifts across salinity groups and predicted metabolic functions

To identify salinity-driven shifts in community composition, the distribution of dominant phyla was compared between brackish and hypersaline microbial mat communities (Supplementary Figure S2). Consistent with the site-level taxonomic patterns (Figure 5), Pseudomonadota was the dominant phylum in both salinity groups, reaching its highest average abundance in hypersaline environments, while Bacteroidota represented the second most abundant phylum and was slightly enriched in brackish habitats. Several phyla associated with anaerobic metabolisms and complex biogeochemical processes, including Desulfobacterota, Verrucomicrobiota, Chloroflexota, Cyanobacteriota, Planctomycetota, Acidobacteriota, and Deinococcota, were consistently enriched in brackish environments. In contrast, hypersaline habitats showed higher relative abundances of Bacillota, Campylobacterota, Halobacteriota, Atribacterota, Nanoarchaeota, and Nanohaloarchaeota, indicating a shift toward halophilic and archaeal-dominated assemblages under elevated salinity conditions. The Z-score distributions further revealed greater taxonomic variability among brackish communities, particularly for Desulfobacterota, Verrucomicrobiota, Chloroflexota, Acidobacteriota, and Planctomycetota, suggesting strong local environmental influences. Conversely, hypersaline environments displayed more consistent dominance of halophilic groups, reflecting stronger environmental filtering under highly saline conditions (Supplementary Figure S2).

**Figure 6:**
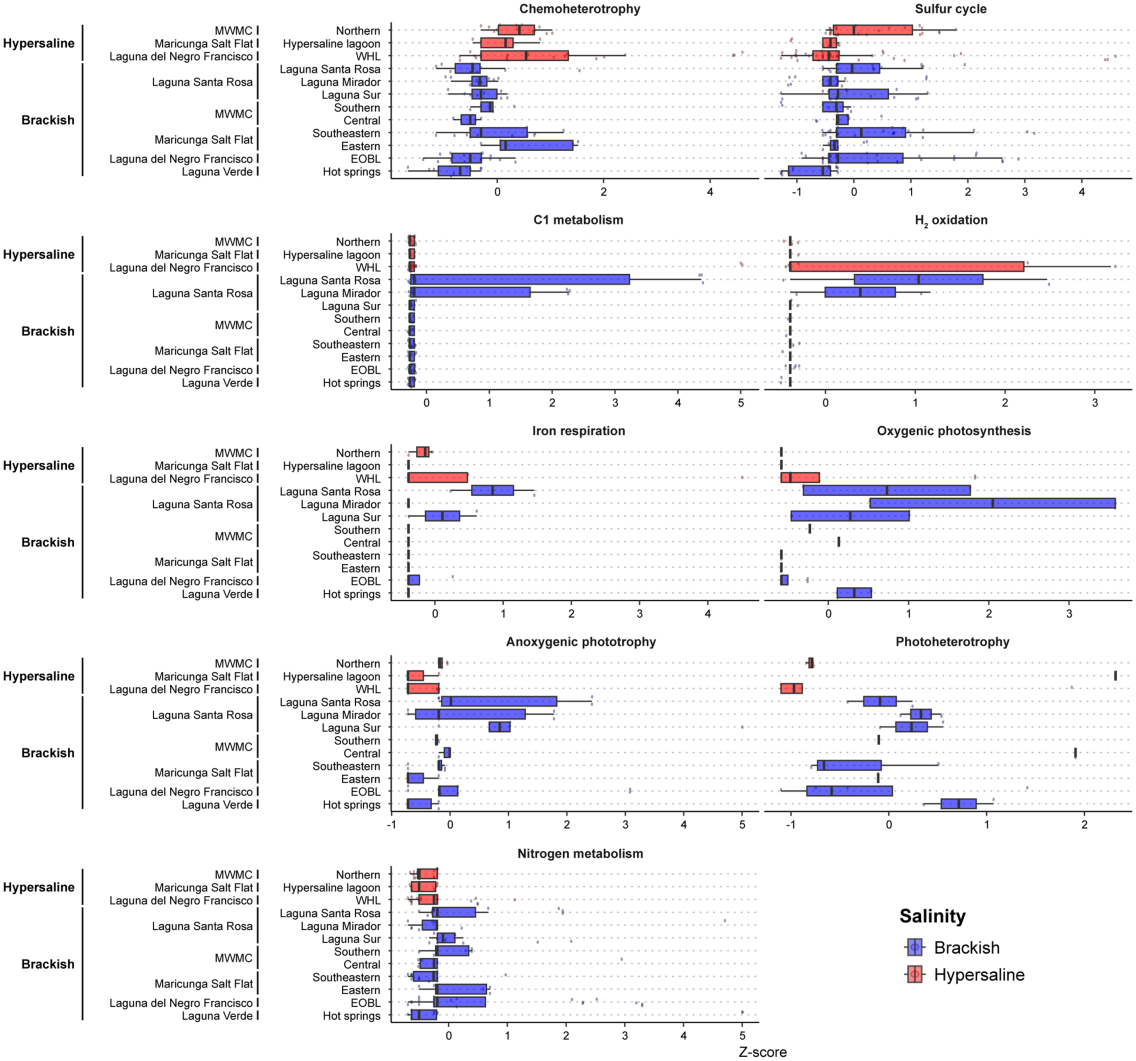
Predicted metabolic profiles of microbial communities inferred using FAPROTAX. Boxplots show the standardized relative abundance (Z-score) of selected metabolic functions across sampling sectors grouped into brackish and hypersaline environments. Colors indicate salinity groups (blue, brackish; red, hypersaline). Boxes represent the interquartile range, center lines indicate the median, and points correspond to individual samples.

To gain insight into the potential biogeochemical functions associated with the dominant microbial taxa, predicted metabolic profiles were generated. The results revealed marked functional heterogeneity among sampling sites and between salinity groups (Figure 6). Chemoheterotrophy was predominantly associated with hypersaline environments, reaching its highest values in the hypersaline lagoon of Maricunga Salt Flat, northern sector of the MWMC, and Western hypersaline lagoon of the Laguna del Negro Francisco. In contrast, oxygenic photosynthesis, anoxygenic phototrophy, photoheterotrophy, and nitrogen metabolism were generally enriched in brackish environments, particularly within Laguna Santa Rosa, where Laguna Sur and Laguna Santa Rosa exhibited the highest relative abundances of these functions. Sulfur-cycle-related metabolisms were widely distributed throughout the hydrological network and occurred in both brackish and hypersaline habitats, representing one of the most consistently detected functional categories across sites. In contrast, C1 metabolism, H₂ oxidation, and iron respiration displayed a more restricted distribution and were concentrated in specific locations. C1 metabolism was primarily associated with Laguna Santa Rosa, whereas H₂ oxidation reached its highest values in the hypersaline sectors of Laguna del Negro Francisco. Iron respiration was detected mainly in Laguna Santa Rosa and the hypersaline lagoon of Laguna del Negro Francisco.

### Shared and site-specific microbial assemblages across the Maricunga hydrological network

Venn analyses revealed substantial taxonomic differentiation among both salinity groups and sampling locations (Supplementary Figure S3; Supplementary Table S3). Considering all detected ASVs, only 206 taxa were shared between brackish and hypersaline environments, whereas 838 and 330 ASVs were exclusive to brackish and hypersaline habitats, respectively. Thus, shared taxa represented only 15% of the total diversity detected across both salinity groups. When increasingly stringent abundance thresholds were applied, the proportion of shared taxa decreased further. At abundances >0.1%, only 197 ASVs were shared, whereas 776 and 300 ASVs were exclusive to brackish and hypersaline environments, respectively. Under the most restrictive threshold (>1%), only 47 ASVs remained shared, while 209 and 86 ASVs were unique to brackish and hypersaline communities. These results indicate that the dominant members of microbial mat communities are largely habitat-specific and that salinity exerts a strong influence on community composition. Site-level comparisons revealed a similar pattern. Laguna Santa Rosa harbored the highest number of unique ASVs across all abundance thresholds, while the MWMC and the Maricunga Salt Flat shared a substantial fraction of taxa with Laguna Santa Rosa. In contrast, Laguna Verde and Laguna del Negro Francisco maintained a larger proportion of exclusive ASVs, even after applying abundance filters, indicating a greater degree of community specialization. Notably, the number of shared taxa progressively decreased as abundance thresholds increased, suggesting that widespread taxa are primarily represented by low-abundance members, whereas dominant taxa tend to be restricted to specific environments (Supplementary Figure S3).

**Figure 7:**
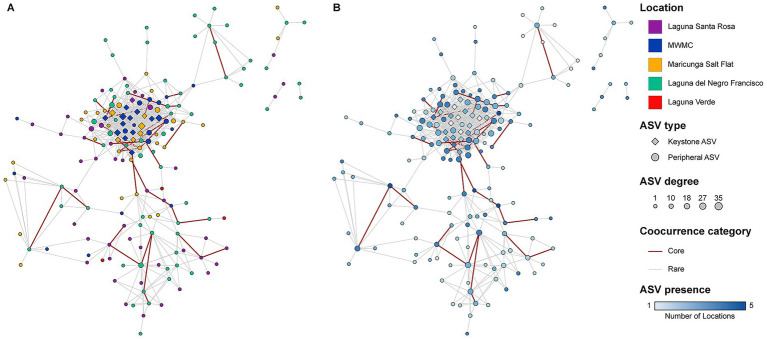
Microbial co-occurrence network inferred from 16S rRNA gene amplicon data. Nodes represent individual ASVs and edges represent significant positive associations between ASVs. **(A)** Network colored according to the location in which each ASV was detected. Diamonds indicate keystone ASVs, whereas circles represent peripheral ASVs. Red edges correspond to associations among core ASVs, and gray edges represent associations involving rare ASVs. **(B)** The same network colored according to ASV occurrence across sampling locations. Node size is proportional to ASV degree (number of connections), and the color gradient indicates the number of ecosystems in which each ASV was detected.

Co-occurrence network analysis further supported the existence of both shared and site-specific community components (Figure 7). Consistent with the Venn analyses, which identified a relatively small proportion of ASVs shared among habitats, the network was structured into several interconnected modules reflecting both hydrological connectivity and local environmental differentiation. Taxa associated with Laguna Santa Rosa, the MWMC, and the Maricunga Salt Flat occupied overlapping regions of the network and formed the largest and most connected cluster, largely dominated by the same major phyla and orders identified in the taxonomic analyses, particularly members of Pseudomonadota and Bacteroidota. In contrast, ASVs associated with Laguna Verde and Laguna del Negro Francisco were more frequently distributed toward the periphery of the network, although maintaining connections with the central modules. Keystone ASVs, identified by diamond-shaped nodes, were distributed throughout the network and were predominantly located at the interface between major clusters, linking different modules and highly connected regions (Figure 7A; Supplementary Table S4). When the same network was visualized according to ASV presence across sampling locations (Figure 7B), a clear gradient in ASV distribution became evident. A large proportion of nodes corresponded to ASVs restricted to a single location, whereas progressively fewer ASVs were shared for all five monitored ecosystems. Notably, many of the keystone ASVs were associated with ASVs present in multiple locations rather than with location-specific taxa. Several of these highly connected keystone nodes occurred within the central regions of the network, and in contrast, location-restricted ASVs were more frequently associated with peripheral network regions and smaller subnetworks. Together, these patterns indicate that the network is composed of a large number of site-specific ASVs embedded within a smaller set of highly connected and geographically widespread taxa. This pattern suggests that a relatively small subset of shared microorganisms contributes disproportionately to network cohesion and connectivity across the hydrological system. Together, the progressive reduction in shared ASVs with increasing abundance thresholds and the distribution of keystone taxa within the network indicate that microbial communities are composed of a highly specialized habitat-specific component superimposed on a smaller but interconnected regional core microbiome. While environmental filtering promotes the establishment of distinct local assemblages, shared and highly connected taxa appear to maintain ecological linkages among hydrologically connected environments.

## Discussion

### Salinity filtering and site-specific drivers of microbial community assembly

The environmental and diversity analyses indicate that microbial community assembly across the studied systems is governed by the interaction between regional environmental filtering and local habitat conditions. Salinity emerged as the only physicochemical variable significantly associated with community variation, supporting its role as an important ecological filter in these high-altitude saline ecosystems. However, the stronger explanatory power of site identity, together with the significant differences in Shannon diversity among locations and the RDA clustering patterns, demonstrates that salinity alone does not fully explain microbial community organization. This pattern is reflected in the taxonomic composition of microbial mats. Although Pseudomonadota and Bacteroidota dominated most communities throughout the system, marked site-specific shifts were observed among secondary phyla. Laguna del Negro Francisco exhibited the strongest taxonomic turnover between its brackish and hypersaline sectors, while hypersaline crusts from the Maricunga Salt Flat and northern MWMC were characterized by the enrichment of Halobacteriota, highlighting the increasing importance of archaeal communities under extreme salinity. In contrast, the overlap observed among Laguna Santa Rosa, MWMC, and the Maricunga Salt Flat suggests that hydrological connectivity contributes to the persistence of a shared microbial component despite environmental heterogeneity. Notably, differences among ecosystems were primarily associated with changes in community composition rather than reductions in diversity, indicating that environmental gradients promote taxonomic replacement and ecological specialization rather than simple diversity loss. Comparable gradient-driven patterns have been documented in other athalassohaline systems of the Chilean Altiplano, where small-scale hydrochemical heterogeneity is associated with pronounced changes in microbial community composition (Paquis et al., 2023; Pardo-Esté et al., 2023). Similarly, studies in Salar de Atacama microbial mats have shown that salinity and geochemical conditions act as major drivers of both taxonomic and functional differentiation (Castro-Severyn et al., 2021; Kurth et al., 2021). Nevertheless, our results suggest that within the studied area, local habitat identity exerts an influence comparable to or greater than that of salinity alone, highlighting the importance of hydrological connectivity and ecosystem-specific environmental conditions in shaping microbial biogeography across high-altitude saline landscapes. By integrating interconnected lagoons, wetlands, saline crusts, and geothermal habitats within a regional environmental framework, this study provides a baseline for understanding microbial diversity and community assembly across the three sub-basins, systems currently exposed to increasing climatic and anthropogenic pressures throughout the Atacama–Puna Plateau.

### Predicted metabolic profiles and a core microbiome across salinity regimes

Across the Maricunga studied area, the dominance of Pseudomonadota and Bacteroidota reflects a common taxonomic signature of high-altitude saline ecosystems, where metabolically versatile heterotrophs frequently dominate microbial mats and sediments (Dorador et al., 2018; Castro-Severyn et al., 2021; Kurth et al., 2021; Osman et al., 2021; Borsodi et al., 2022). However, salinity promoted clear taxonomic differentiation, with brackish habitats enriched in Desulfobacterota, Chloroflexota, Cyanobacteriota, Planctomycetota, and Verrucomicrobiota, whereas hypersaline environments favored halophilic archaeal lineages such as Halobacteriota, Nanoarchaeota, and Nanohaloarchaeota. Similar archaeal enrichment has been reported in hypersaline mats and microbialites from the Salar de Atacama, where elevated salinity and evaporation promote haloarchaeal-driven trophic networks and osmoadaptive strategies (Dorador et al., 2010, 2013; Stivaletta et al., 2011; Ventosa et al., 2015; Fernandez et al., 2016; Farías et al., 2017).

These taxonomic differences were accompanied by predicted functional differentiation. Brackish environments, particularly Laguna Santa Rosa, showed higher representation of photosynthetic and nitrogen-related metabolisms, whereas hypersaline sectors of Laguna del Negro Francisco and the Maricunga Salt Flat exhibited the strongest chemoheterotrophic signatures. Sulfur-related metabolisms remained abundant across nearly all ecosystems, highlighting sulfur transformations as a key ecological process throughout the studied environments. Similar functional partitioning has been described in microbial mats from the Salar de Atacama, where increasing salinity promotes the replacement of phototrophically supported communities by metabolically specialized assemblages dominated by halophilic microorganisms (Castro-Severyn et al., 2021; Kurth et al., 2021). The distinctive representation of Planctomycetota, Chloroflexota, and Cyanobacteriota in Laguna Verde further supports the existence of specialized microbial consortia associated with geothermal and geochemically unique habitats (Vignale et al., 2021; Borsodi et al., 2022; Osman et al., 2024).

The Venn analyses and co-occurrence network revealed that environmental filtering and hydrological connectivity jointly shape microbial community organization. Although only 15% of detected ASVs were shared between brackish and hypersaline environments, the modular architecture of the co-occurrence network indicates strong species sorting along physicochemical gradients, particularly salinity, which has been identified as a major determinant of microbial community structure across diverse ecosystems (Lozupone and Knight, 2007; Hanson et al., 2012). Nevertheless, several highly connected keystone ASVs were distributed across multiple ecosystems and occupied central positions within the network, linking the largest cluster formed by Laguna Santa Rosa, the MWMC, and the Maricunga Salt Flat with the more peripheral assemblages of Laguna Verde and Laguna del Negro Francisco. These shared ASVs likely represent a regional microbial pool maintained through hydrological connectivity, whereas unique ASVs reflect habitat-specific lineages selected by local environmental conditions. Together, these findings indicate that salinity drives both taxonomic and functional specialization, while a smaller set of highly connected microorganisms maintains ecological cohesion across the studied environments. The coexistence of habitat-specific assemblages and a regional core microbiome emerge as a defining feature of these high-altitude saline systems and provides a robust ecological baseline for future metagenomic and metatranscriptomic investigations of microbial adaptation and biogeochemical cycling in the Atacama-Puna region.

## Conclusion

By integrating environmental gradients, microbial diversity, taxonomic composition, predicted metabolic functions, and co-occurrence networks, this study reveals that the Maricunga Salt Flat, MWCM, Laguna Santa Rosa, Laguna del Negro Francisco and Laguna Verde functions as a connected microbial macrosystem. Salinity promotes strong taxonomic and functional specialization, while hydrological connectivity sustains a regional pool of shared microorganisms and keystone taxa that maintains ecological cohesion across the studied environments. The identification of both habitat-specific assemblages and a regional core microbiome represent a key ecological insight for understanding microbial organization in high-altitude saline ecosystems. Beyond their extreme physicochemical conditions, the wetlands, lagoons, geothermal habitats, and evaporitic environments of the studied Atacama-Puna systems constitute unique natural laboratories for exploring microbial adaptation, community assembly, and biogeochemical cycling under polyextreme conditions. As one of the largest and environmentally most relevant salt-flat systems of the Chilean Altiplano, Maricunga Salt Flat, MWMC, Laguna Santa Rosa, Laguna del Negro Francisco and Laguna Verde provide an exceptional opportunity to investigate how microbial communities respond to salinity gradients, geothermal influence, and hydrological connectivity across a single integrated landscape.

As climate variability and anthropogenic pressures associated with water use and mineral extraction continue to intensify across the Atacama–Puna Plateau, the ecological baseline established here becomes increasingly valuable. Future metagenomic and metatranscriptomic analyses will allow direct characterization of the metabolic potential and activity of these microbial communities, providing deeper insights into the ecological functions, adaptive mechanisms, and biogeochemical processes that sustain ecosystem resilience in one of the most extreme and rapidly changing environments on Earth.

## Data Availability

The datasets generated during the current study are available in the NCBI repository under the BioProject accession number PRJNA1458444.

## Footnotes

1
https://hbiostat.org/r/hmisc/

## Supplementary material

Supplementary Figure S1 Heatmap of the relative abundance of taxonomic orders across sampling sites. The heatmap shows the relative abundance of taxonomic orders, expressed as log₂ (relative abundance (%) + 1), in samples collected from Laguna Santa Rosa, the Maricunga Wetland Main Channel (MWMC), the Maricunga Salt Flat, Laguna del Negro Francisco, and the geothermal springs of Laguna Verde. Columns represent individual samples, rows represent taxonomic orders, and color intensity corresponds to relative abundance according to the scale shown.

Supplementary Figure S2 Comparison of phylum-level taxonomic composition across salinity categories. The left panel shows the mean relative abundance of each phylum, expressed as log₁₀ (relative abundance (%) + 1), in brackish and hypersaline environments. The right panel displays the standardized relative abundance (Z-score) of each phylum across individual samples. Colors indicate salinity category (blue: brackish; red: hypersaline). Boxplots represent the median and interquartile range, and points correspond to individual samples.

Supplementary Figure S3 Venn diagrams show the distribution of amplicon sequence variants (ASVs) among salinity groups and ecosystems. The left panels compare shared and unique ASVs between brackish and hypersaline environments, whereas the right panels show ASV sharing among Laguna Santa Rosa, the Maricunga Wetland Main Channel (MWMC), the Maricunga Salt Flat, Laguna del Negro Francisco, and Laguna Verde. Top panels include all detected ASVs, middle panels include ASVs with relative abundances greater than 0.1%, and bottom panels include ASVs with relative abundances greater than 1%. Numbers within each section indicate the count of unique or shared ASVs. Circle color intensity corresponds to the number of ASVs represented in each category, as indicated by the associated color scales.

Supplementary Table S1 Physicochemical characteristics of the sampling sites. Geographic location, sampling sector, sample identifier, and physicochemical characteristics of all microbial sampling sites included in this study. Environmental variables measured at each site included temperature (°C), pH, conductivity (mS cm⁻¹), total dissolved solids (TDS, ppt), salinity (psu), and dissolved oxygen (ppm). Standardized values used for multivariate analyses are also provided.

Supplementary Table S2 Forward selection analyses identifying environmental variables associated with microbial community composition. Based on Bray–Curtis dissimilarities of Hellinger-transformed 16S rRNA gene data, the table presents models including only environmental variables and models including site as an additional explanatory factor. Environmental variables evaluated were salinity (ppm), temperature (°C), pH, and dissolved O2 (ppm). Adjusted R² and adjusted *p*-values are reported for each model.

Supplementary Table S3 Distribution of ASVs across sampling locations and salinity categories. The table summarizes the number of amplicon sequence variants (ASVs) detected in one to five sampling locations and the number of unique and shared ASVs between brackish and hypersaline environments. Values are reported for all detected ASVs, ASVs with relative abundances >0.1%, and ASVs with relative abundances >1%.

Supplementary Table S4 Taxonomic identity of keystone ASVs identified in the co occurrence network analysis. The table presents the taxonomic classification of the keystone amplicon sequence variants (ASVs) identified in the co-occurrence network, including their assignment from phylum to genus, when available.
